# Integrated Approach Using Intuitionistic Fuzzy Multicriteria Decision-Making to Support Classifier Selection for Technology Adoption in Patients with Parkinson Disease: Algorithm Development and Validation

**DOI:** 10.2196/57940

**Published:** 2024-10-22

**Authors:** Miguel Ortiz-Barrios, Ian Cleland, Mark Donnelly, Muhammet Gul, Melih Yucesan, Genett Isabel Jiménez-Delgado, Chris Nugent, Stephany Madrid-Sierra

**Affiliations:** 1 Department of Productivity and Innovation, Universidad de la Costa CUC, 58th street #55-66, Barranquilla, 080002, Colombia, 57 3007239699; 2 School of Computing, Ulster University, Belfast, United Kingdom; 3 School of Transportation and Logistics, Istanbul University, Istanbul, Turkey; 4 Department of Emergency Aid and Disaster Management, Munzur University, Munzur, Turkey; 5 Department of Industrial Engineering, Institución Universitaria de Barranquilla, Barranquilla, Colombia

**Keywords:** Parkinson disease, technology adoption, intuitionistic fuzzy analytic hierarchy process, intuitionistic fuzzy decision-making trial and evaluation laboratory, combined compromise solution

## Abstract

**Background:**

Parkinson disease (PD) is reported to be among the most prevalent neurodegenerative diseases globally, presenting ongoing challenges and increasing burden on health care systems. In an effort to support patients with PD, their carers, and the wider health care sector to manage this incurable condition, the focus has begun to shift away from traditional treatments. One of the most contemporary treatments includes prescribing assistive technologies (ATs), which are viewed as a way to promote independent living and deliver remote care. However, the uptake of these ATs is varied, with some users not ready or willing to accept all forms of AT and others only willing to adopt low-technology solutions. Consequently, to manage both the demands on resources and the efficiency with which ATs are deployed, new approaches are needed to automatically assess or predict a user’s likelihood to accept and adopt a particular AT before it is prescribed. Classification algorithms can be used to automatically consider the range of factors impacting AT adoption likelihood, thereby potentially supporting more effective AT allocation. From a computational perspective, different classification algorithms and selection criteria offer various opportunities and challenges to address this need.

**Objective:**

This paper presents a novel hybrid multicriteria decision-making approach to support classifier selection in technology adoption processes involving patients with PD.

**Methods:**

First, the intuitionistic fuzzy analytic hierarchy process (IF-AHP) was implemented to calculate the relative priorities of criteria and subcriteria considering experts’ knowledge and uncertainty. Second, the intuitionistic fuzzy decision-making trial and evaluation laboratory (IF-DEMATEL) was applied to evaluate the cause-effect relationships among criteria/subcriteria. Finally, the combined compromise solution (CoCoSo) was used to rank the candidate classifiers based on their capability to model the technology adoption.

**Results:**

We conducted a study involving a mobile smartphone solution to validate the proposed methodology. Structure (F5) was identified as the factor with the highest relative priority (overall weight=0.214), while adaptability (F4) (D-R=1.234) was found to be the most influencing aspect when selecting classifiers for technology adoption in patients with PD. In this case, the most appropriate algorithm for supporting technology adoption in patients with PD was the A3 - J48 decision tree (*M*_3_=2.5592). The results obtained by comparing the CoCoSo method in the proposed approach with 2 alternative methods (simple additive weighting and technique for order of preference by similarity to ideal solution) support the accuracy and applicability of the proposed methodology. It was observed that the final scores of the algorithms in each method were highly correlated (Pearson correlation coefficient >0.8).

**Conclusions:**

The IF-AHP-IF-DEMATEL-CoCoSo approach helped to identify classification algorithms that do not just discriminate between good and bad adopters of assistive technologies within the Parkinson population but also consider technology-specific features like design, quality, and compatibility that make these classifiers easily implementable by clinicians in the health care system.

## Introduction

### Background

Advances in the economy, health care, science, and technology have significantly influenced demographics. Between 2000 and 2019, global average life expectancy increased by over 6 years to 73.4 years; however, healthy life expectancy has not kept pace [1]. Consequently, the years spent living with illness or disease have increased, with approximately 1 in 3 adults suffering from multiple chronic conditions, and 3 in 4 older adults living with 1 or more chronic conditions [2]. This has added unsustainable pressure on society’s ability to provide long-term economic care, promoting a renewed drive for innovative treatment.

One initiative has been to seek efficiencies in health care delivery through prescribing assistive technologies (ATs). ATs typically support health care outside traditional settings, aiding in remote monitoring of conditions, thereby promoting the independence of individuals and caregivers. Older users, however, who tend to be less familiar with technology advancements, remain hesitant to readily adopt ATs as a long-term, low-cost replacement for human care. Consequently, low acceptance rates, along with the requirement to update prescribed ATs as a condition evolves, remain a significant challenge to widespread adoption [3].

One mitigation is to preassess adoption likelihood so that the appropriate solutions are deployed, decommissioned, and replaced accordingly over time. A research challenge exists to appropriately identify and develop automated algorithms that can assess adoption likelihood. This paper investigates this challenge and extends our previous work, identifying the most appropriate classification algorithms to support AT assessment [4,5]. The novelty of this study also lies in the use of an integrated intuitionistic fuzzy multicriteria decision-making (MCDM) approach to dealing with this problem. This approach addresses uncertainty better with the nonmembership function [6], which helps better define the evaluations of decision makers [7], and minimizes information loss in operations with fuzzy numbers [8]. Specifically, we used intuitionistic fuzzy analytic hierarchy process (IF-AHP) to estimate initial criteria weights, intuitionistic fuzzy decision-making trial and evaluation laboratory (IF-DEMATEL) to evaluate interrelations among criteria, and combined compromise solution (CoCoSo) to rank classifiers. The study uncovered factors influencing the design of algorithms that can accurately prescribe AT. The results highlight scalability, adaptability, and performance as key criteria alongside ease of interpretation for confident deployment and the use of transparent, white-box algorithms to enhance usability and acceptance. The paper presents the finding using a case study considering technology adoption among patients with Parkinson disease (PD), which is a leading chronic condition affecting approximately 10 million people, with the majority of symptoms typcially developing after age 50 [9].

In this paper, we begin by presenting related works to highlight the opportunities and challenges in this research domain, then describe the proposed methodological approach. Next, we present and critique the main findings of our work, and finally consolidate these observations toward summarizing the main scientific implications evidenced.

### Review of the Literature

Statistical and machine learning (ML) approaches are increasingly promising in technology adoption modeling research. In particular, ML is vital in advancing and validating theoretical frameworks of technology adoption and improving their predictive power.

The most popular theories for technology adoption are the Technology Acceptance Model (TAM) and the Unified Theory of Acceptance and Use of Technology (UTAUT) [10]. Both TAM and UTAUT suggest that technology use is impacted by an individual’s behavioural intention to use it. In the TAM, a person’s attitude to technology, determined by perceived usefulness and perceived ease of use, is used to measure intention to use [11]. UTAUT builds on this, in addition to other theoretical frameworks. In UTAUT, four constructs impacting intention to use are considered: (1) performance expectancy, (2) effort expectancy, (3) social influence, and (4) facilitating conditions. UTAUT additionally considers constructs of age, gender, voluntariness, and experience of use to temper expectations of intention to use by the individual.

Historically, researchers in technology adoption have considered 3 elements when modeling adoption: users, technology, and environment. They have constructed these elements within several frameworks mentioned above. Although frameworks such as these have made significant inroads in furthering our understanding of technology adoption, they are not without limitations. Both TAM and UTAUT have been criticized for being overly simplistic and focusing on a narrow perspective of individuals’ beliefs, perceptions, and usage intention. Additionally, several studies have highlighted that these theories no longer contribute new knowledge or understanding to technology adoption. Therefore, new ways of understanding technology adoption are required [11].

Recently, technology adoption researchers have highlighted an additional limitation of frameworks, including TAM and UTAUT. Specifically, these models have been developed focusing on explanatory and causal modeling techniques. This may overlook the nonlinearity and influence of technology-specific features such as design, quality, or compatibility [12]. With the rise of the availability of discrete data sources, such as that generated using digital health applications, there has been greater interest from the research community in data-driven approaches for technology adoption. ML approaches to technology adoption can be broadly split into 2 groups: predictive modeling and explanatory modeling [12]. Predictive modeling seeks to predict actual use behavior (adopt or not), while descriptive modeling is focused on the interaction between various constructs that influence the adoption of a specific technology.

As a data-driven modeling technique, an ML methodology empirically predicts targeted output, adopted or not. Although it is possible to combine predictive modeling with explanatory modeling, research has shown that higher accuracy can be achieved using only predictive modeling. The 2 common approaches to developing a predictive model using ML for technology adoption are supervised and unsupervised. In supervised ML, the most common approach is to develop predictive models using classification or regression. Supervised ML models used for technology adoption have included multiple linear regression, support vector machine, multilayer perceptron, random forest, decision tree, or ensemble methods [12,13]. The model’s predictive accuracy is measured by comparing the performance of 1 or more ML algorithms. In contrast, unsupervised ML for technology adoption is developed by applying ML algorithms, typically based on clustering, to gain insight into the factors that inform adoption. To complement and enhance the performance of ML, feature selection techniques can be used to reduce the dimensionality of the data and improve the reliability of the model. Feature selection techniques help to exclude irrelevant factors that have a negligible impact on the model or are redundant.

The Technology Adoption and Usage Tool project aimed to model the adoption of mobile-based reminding solutions by people with dementia and their carers [12]. The project took an iterative approach to model development, using a unique and diverse dataset obtained by recruiting 335 participants. The dataset contained genealogical, medical, and demographic records created by combining data from the Cache County Study on Memory in Aging and the Utah Population Database. Participants were categorized into four groups: 3 types of nonadopter (1=willing but unable, 2=not willing and not able, 3=not willing but able) and 1 adopter group. The study assessed the ability to classify whether an individual would adopt the technology using various ML algorithms. Results showed that including psychosocial and medical history information, the developed adoption model, based on the *k*-nearest neighbors (*k-*NN) algorithm, achieved a prediction accuracy of 99.41% [14]. The study also investigated the effect of feature selection on each algorithm, with information gain used to rank features in terms of discriminating power for classifications.

Ortiz et al [15] proposed a multicriteria decision-making approach for technology adoption modeling for people with dementia. This work applied a fuzzy analytic hierarchy process (FAHP) to estimate the initial weights of criteria and subcriteria. The decision-making trial and evaluation laboratory (DEMATEL) was then used to evaluate the relationship and feedback among criteria. The technique for order of preferences by similarity to ideal solution (TOPSIS) was then used to rank 3 classifiers (*k-*NN, naive Bayes, and decision tree) according to their ability to model technology adoption. Results showed that flexibility and design were the most relevant criteria, with overall weights of 0.235 and 0.260, respectively. Naive Bayes was the most suitable classifier, with a closeness coefficient of 67.7%. It was noted that there was room for further improvement of all models tested in terms of performance and scalability.

As highlighted by the related work, ML adoption models have seen significant improvements since being first developed. These models have been tailored to suit some use cases and technical solutions. The models have also been extended to include a range of constructs and demographics [14,16]. The likelihood of adoption is transient and spans not only the physical product design and characteristics of the individual but also the social settings and channels through the technology implemented and disseminated. Indeed, a user’s perception of technology’s ease of use and usefulness may change over time as the needs, capabilities, and perceptions of the individual and society change and technology capabilities advance.

Indeed, evidence suggests there are substantial benefits to be made for ML-based approaches to technology adoption [17]. Simple regression-based models have a demonstratable ability to predict individuals who are likely to adopt technology with an accuracy of over 90% [14]. Parameters used as input into these models have ranged from sociodemographic information, such as age and education, to measures of prior technology experience and perceived usefulness/ease of use. Increasing input parameters include detailed medical history [14]. It has also been possible, through the inclusion of additional processing steps of selecting features, to refine the adoption model and improve the generalization of the modeling process [14]. Adoption models have been evaluated and chosen solely based on performance (accuracy). There would be a benefit in paying closer attention to other important metrics when selecting a suitable classifier. As different classifiers and selection criteria can be considered for addressing this problem, this paper presents a hybrid MCDM approach to support classifier selection in technology adoption processes involving patients with PD. First, the IF-AHP is implemented to calculate the relative priorities of criteria and subcriteria considering experts’ knowledge and uncertainty. Second, the IF-DEMATEL was applied to evaluate the cause-effect relationships among criteria/subcriteria.

The methodology we propose differs from similar studies in the literature in terms of its theoretical and practical contributions. As a methodological contribution, MCDM methods are integrated. Thanks to MCDM, effective and reliable decisions can be made [18], and complex problems can be solved by breaking them into smaller parts [19]. MCDM is a methodology that guides decision-makers in structuring and solving decision and planning problems involving multiple criteria [20]. Decision-makers use MCDM methods to evaluate possible alternatives and determine how these alternatives affect the decision-making objective [21]. Furthermore, MCDM methods can help the decision-maker determine each criterion’s importance and identify trade-offs between these criteria. Thus, a comparative application can be performed with MCDM methods, and the best alternative solutions can be provided to decision-makers. Although decision-makers use MCDM methods in health care management, such as health care performance assessment [22] measuring the efficiency of hospitals [23], their use in specialized areas, such as health care technology adoption, is rare [24]. As a practical contribution, there is no cure for PD. Although it is impossible to access actual data when selecting AT that will increase the patient’s quality of life, the benefits of ATs vary from patient to patient. Therefore, determining the appropriate classification algorithm also includes vagueness and ambiguity.

In recent years, decision-makers have integrated multiple MCDM methods for complex problems [25]. AHP, DEMATEL, and CoCoSo from MCDM methods were used in the study. AHP does not require complex mathematical calculations used in criteria weighting and allows the decision-maker to focus on each criterion [26]. Since the AHP method could not reflect the uncertainty of the decision-makers, a method named FAHP was developed by using fuzzy logic and AHP together [27]. However, FAHP was also criticized in the literature because it did not express uncertainty. Therefore, the IF-AHP method is more effective in addressing the hesitations of decision-makers [28].

Unlike traditional MCDM methods, DEMATEL offers a more appropriate solution to real-world problems by considering the interactions between criteria [29,30]. Standard DEMATEL may often fail to represent the uncertainty encountered in real-world problems [31]. To overcome this situation, an attempt is made to deal with the uncertainty by integrating DEMATEL with fuzzy logic [32]. IF integration with DEMATEL has been realized. DEMATEL calculation is almost the same as IF-DEMATEL. The most apparent differences are the input data and averaging method [33]. In IF-DEMATEL, decision-makers express their preferences with intutionistic fuzzy sets (IFS). In group information, the intuitionistic fuzzy weighted averaging (IFWA) operator is used [34].

CoCoSo is a method based on the integration of the recently developed weighted sum method and weighted product method [35,36]. CoCoSo provides a more robust solution than traditional MCDM methods [37]. It is integrated with AHP and DEMATEL in an IF environment. With IF-AHP, decision-makers were provided with the ability to express uncertainties better, and a more realistic evaluation was made. Similarly, cause and effect criteria were determined with IF-DEMATEL. Finally, candidate classifiers were ranked according to their transferability index using CoCoSo. Another contribution of the study to the literature is in the validation part. Thanks to the mobile phone app, the proposed methodology has been verified.

This literature review highlights several critical research gaps in technology adoption modeling. First, while statistical and ML approaches, particularly ML, hold great promise in advancing theoretical frameworks of technology adoption and improving their predictive power, there is a need for more nuanced models that account for nonlinear relationships and technology-specific features like design, quality, and compatibility. Additionally, the evaluation of classifiers has traditionally been based solely on performance (accuracy). However, other metrics should be taken into account for a more comprehensive assessment. The proposed MCDM approach offers a promising method for integrating various criteria and subcriteria to make more effective and reliable decisions in technology adoption processes.

To address these research gaps, this study introduces a hybrid MCDM approach to aid in selecting classifiers for technology adoption processes, specifically those involving patients with PD. First, considering both expert knowledge and uncertainty, the IF-AHP was utilized to determine the relative priorities of criteria and subcriteria. Next, the IF-DEMATEL was used to assess the cause-effect relationships among these criteria and subcriteria. Last, the CoCoSo was used to rank the potential classifiers based on their effectiveness in modeling technology adoption. A mobile smartphone solution case study was conducted to validate the proposed methodology.

### A Brief Criticism and Gap Analysis in Technology Adoption Literature

The literature review explores the evolution and limitations of technology adoption modeling, emphasizing the growing significance of statistical and ML approaches. Although foundational theories like the TAM and UTAUT have shaped understanding by focusing on individual beliefs and perceptions, they are criticized for their simplicity and narrow focus on individuals’ beliefs and intentions as well as oversimplification and neglect of broader contextual factors. Besides, they often fail to account for the complex interactions between users, technology, and the environment, as well as nonlinear relationships and technology-specific features like design, quality, and compatibility. Furthermore, these traditional theories no longer provide new insights into technology adoption, necessitating new approaches. Their reliance on explanatory and causal modeling techniques overlooks the potential of unobtrusive data sources and the inherent nonlinearity in adoption processes.

Recent research highlights the need for more sophisticated models that explain nonlinear relationships and technology-specific features such as design and compatibility. ML methods, categorized into predictive and explanatory modeling, offer promising avenues for enhancing predictive accuracy, although challenges remain in evaluating models beyond traditional metrics like accuracy. Current literature gaps include the overemphasis on performance metrics, neglecting other important evaluation criteria.

Integrating MCDM methods addresses some of these challenges by considering criteria such as flexibility and user preference, thus providing a more comprehensive approach to classifier selection. Empirical validations, such as the successful prediction of technology adoption among patients with dementia using ML algorithms, underscore the potential and the necessity for ongoing refinement and ethical considerations in technology adoption research. Recent studies suggest the usage of MCDM methods in the selection of ML classifiers to address these limitations. Methods like the IF-AHP and IF-DEMATEL can evaluate a broader range of criteria, offering a more comprehensive understanding of technology adoption. Moreover, an important MCDM method such as CoCoSo successfully ranks the classifiers based on their effectiveness in modeling technology adoption. Thus, a study like the current attempt is needed to validate these methodologies and explore their application in different technological contexts, such as health care technology adoption, where individual needs and preferences play a crucial role in adoption decisions.

## Methods

### Overview

**Figure 1.**
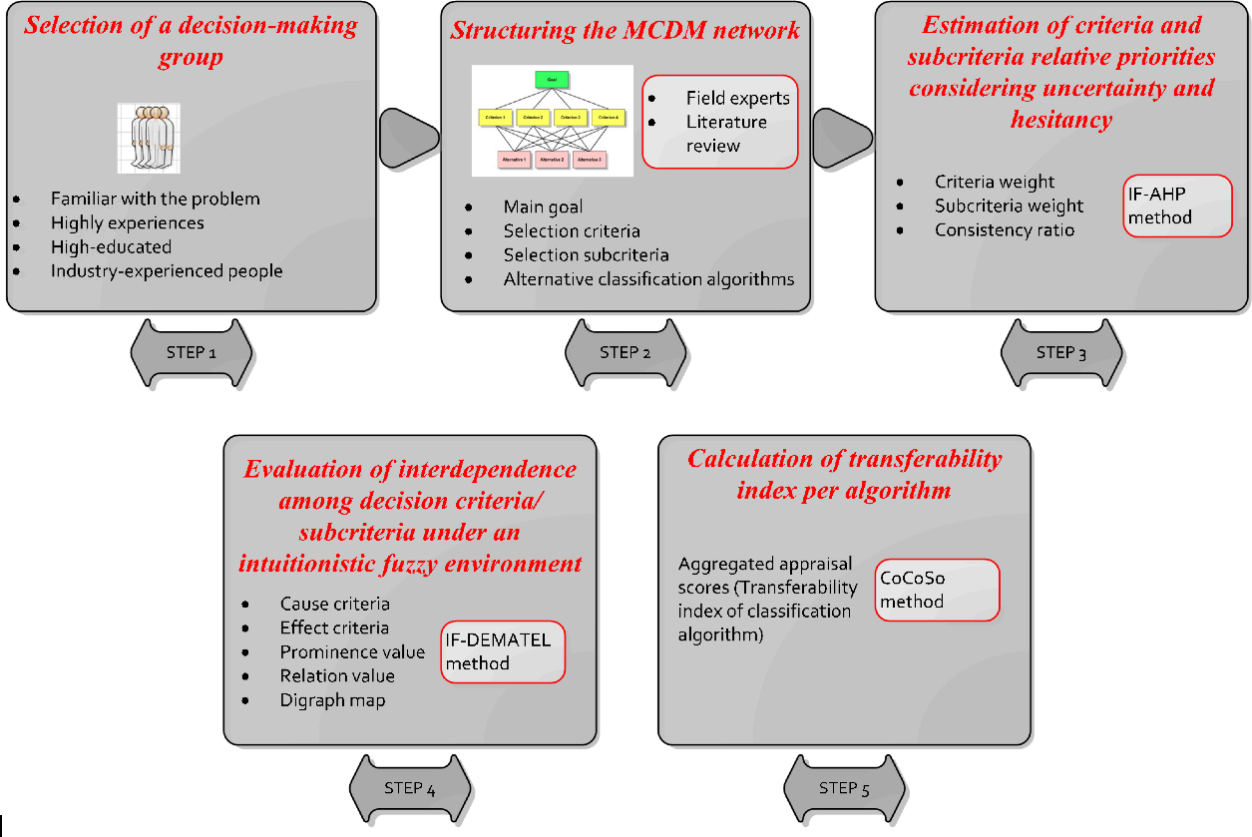
Flow chart of the 5-step intuitionistic fuzzy MCDM approach. CoCoSo: combined compromise solution; IF-AHP: intuitionistic fuzzy analytic hierarchy process; IF-DEMATEL: intuitionistic fuzzy decision-making trial and evaluation laboratory; MCDM: multicriteria decision-making.

A 5-step intuitionistic fuzzy MCDM approach is proposed to support classifier selection in technology adoption for people with PD (Figure 1). The validation process considers a mobile smartphone solution entailing 4 intervention categories: tipping, memory, walking, and voice. An intricate explanation of this framework is provided below.

### Step 1: Selection of a Decision-Making Group

This step is about establishing a decision-making team that compares criteria and subcriteria in both the IF-AHP and IF-DEMATEL phases. The team is expected to be familiar with both the criteria set in the structure of the problem and the alternative algorithms that are likely to be evaluated. Although determining the transferability indexes of the algorithms is the task of the CoCoSo model, this team must know the general outline of the problem. It is recommended that the experts enrolled in this team be selected from experienced, highly educated, and industry-experienced people.

### Step 2: Structuring the MCDM Network

What is meant by the MCDM network, of course, is the decision structure formed by the selection criteria and subcriteria. This network also includes the main goal and alternative classification algorithms in this network. At this stage, the literature and the opinions of field experts were used to create decision criteria. Since the most crucial step in revealing the problem is establishing this MCDM network, it should be kept in mind that ignoring a criterion or subcriterion that affects the selection process will affect the final decision and may lead to a wrong selection.

### Step 3: Estimating Criteria and Subcriteria Relative Priorities Considering Uncertainty and Hesitancy

This section is about determining the relative importance levels of criteria and subcriteria under the uncertainty of the decision process and the decision-maker’s hesitation. At this point, the advantages of membership and nonmembership features that intuitionistic fuzzy sets suggest for high uncertainty and hesitation in decision-making emerge. At the same time, its combination with the AHP algorithm provides both the individual advantages of the 2 concepts and the integrated advantages. Although AHP is easy to use and widespread in the literature, it falls short in responding to the hesitant structure in decision-making, and this gap can be remedied with IF-AHP. We aim to make pairwise comparisons of the expert group established in the IF-AHP phase and to find the relative importance of the criteria and subcriteria by using the IF-AHP algorithm.

### Step 4: Evaluation of Interdependence Among Decision Criteria/Subcriteria Under an Intuitionistic Fuzzy Environment

The reason for using the DEMATEL (under intuitionistic fuzzy environment) method in this triple structure (as IF-AHP, IF-DEMATEL, and CoCoSo) is to determine the relationship between the criteria and subcriteria (of which their weights are obtained via IF-AHP algorithm in the first phase) and focus on the strength and presence of feedback. In other words, it is determining which criteria/subcriteria are the cause and which is the effect criterion regarding classifier selection decision-making in technology adoption for people with PD.

### Step 5: Calculation of Transferability Index Per Algorithm

In the final phase of the triple structure, the CoCoSo MCDM method is used to compute each transferability index of classification algorithms used in technology adoption for people with PD. This index value will measure the algorithm’s capability to model the technology adoption. It is good to note that IF-CoCoSo was not proposed for this case, considering that indicators’ values of subcriteria are known and available. IF-CoCoSo is typically adopted when there is imprecise knowledge or a lack of data [38]. In this line, the crisp CoCoSo is enough to derive the transferability index without loss of information.

### IF-AHP Algorithm

The IF-AHP algorithm is an MCDM approach that integrates intuitionistic fuzzy set logic into the AHP algorithm. In addition to denoting the uncertainty and vagueness of human thought regarding the technology adoption context, the IF logic is used in this case to represent the knowledge level of experts, which may vary from one to the other, hinging upon educational background and experience [33,34,39]. This latter aspect cannot be typified by type-2 fuzzy nor hesitant fuzzy sets, which is the reason why they were discarded from this application. To explain in detail how the IF-AHP algorithm works, it is helpful to present some notations (basic math operations, defuzzification, aggregation operators, etc) about this fuzzy set extension. Atanassov [39] was the first to propose this fuzzy set extension. After being presented, it has been applied to many decision problems in many different industries [40]. There are 2 functions for this type of fuzzy set: membership and nonmembership. The sum of the degrees of membership and nonmembership is always equal to 1. The step-by-step flow of the IF-AHP algorithm is as follows:

An intuitionistic fuzzy set “I” is defined by Equation 1 [41-43]:


(1) $$I=\left\{x,\;I\left(\mu_{I}\left(x\right),v_{I}\left(x\right)\right)|x\in X\right\}$$

where X is a set in a universe of discourse and $\mu_{I}(x)$ refers to the degree of membership, $v_{I}(x)$ refers to the degree of nonmembership, and $\pi_{I}(x)$ refers to the degree of lack of knowledge for each x∈X:


(2) $${0\leq \mu }_{I}\left(x\right)+v_{I}(x)\leq 1$$

(3) $$\pi_{I}\left(x\right)=1-\mu_{I}\left(x\right)-v_{I}\left(x\right),x\in X$$

One of the critical aspects of intuitionistic fuzzy set notation is defuzzification. Anzilli and Facchinetti [44] and Ocampo and Yamagishi [43] proposed and used a different defuzzification method as in Equations 4 and 5.


(4) $$C_{\varphi }\left(I\right)=\left\{\left\langle x,\mu_{I}\left(x\right)+\varphi \pi_{I}\left(x\right),v_{I}\left(x\right)+\left(1-\varphi \right)\pi_{I}\left(x\right)\right\rangle ,x\epsilon X\right\}\;with\;\varphi \epsilon \left[0,1\right]$$

(5) $$\mu_{\varphi }\left(x\right)=\mu_{I}\left(x\right)+\varphi \pi_{I}\left(x\right)$$

$C_{\varphi }(I)$ is a defuzzification operator defined in Equation 4 under a usual fuzzy subset with the membership function given by Equation 5. Mostly, φ=0.5 is a solution of the minimization problem $\min_{\varphi \epsilon \left[0,1\right]}d\left(C_{\varphi }\left(I\right),\;I\right)$. Here, d refers to the Euclidian distance. With φ=0.5, a membership function $\mu \left(x\right)=\frac{1}{2}\left(1+\mu_{I}\left(x\right)-v_{I}\left(x\right)\right)$ characterizes the fuzzy set $C_{0.5}\left(I\right)$.

We benefitted from the studies of Karacan et al [45] and Abdullah and Najib [46] in determining the triangular intuitionistic fuzzy numbers-based preference scale. The IF-AHP algorithm we used in this study is processed as follows:

The first step starts with determining the decision criteria and subcriteria regarding the selection of classification algorithms supporting effective AT allocation, fostering independent living while reducing the economic and social burden faced by patients with PD and their carers.

The main argument of IF-AHP as “pairwise comparisons” is made in the second step, following the scale of Karacan et al [45]. The scale has 5 points: “much more importance” (0.33, 0.27, 0.40), “more importance” (0.13, 0.27, 0.60), “equal importance” (0.02, 0.18, 0.80), “less importance” (0.27, 0.13, 0.60), and “much less importance” (0.27, 0.33, 0.40). The ternaries are in the form of ($\mu_{I}\left(x\right),v_{I}\left(x\right),\pi_{I}\left(x\right)$), denoting belongingness (affirmation/agreement), nonbelongingness (negation/disagreement), and lack of knowledge (indeterminacy/abstention) levels [47].

Another important argument of decision-making problems is performed in this step. The assignment of a coefficient for experts who assessed the criteria and subcriteria is fulfilled. The triangular intuitionistic fuzzy scale proposed by Boran et al [48] is used. It is a 5-point scale with “very important” (0.90, 0.05, 0.05), “important” (0.75, 0.20, 0.05), “medium important” (0.50, 0.40, 0.10), “unimportant” (0.25, 0.60, 0.15), and “very unimportant” (0.10, 0.80, 0.10). Assignment of a weight to a member of the expert team is performed by Equation 6.


(6) $$\omega_{k}=\frac{\left(\mu_{k}+\pi_{k}\left(\mu_{k}/\left(\mu_{k}+v_{k}\right)\right)\right)}{\sum_{k=1}^{t}\left(\mu_{k}+\pi_{k}\left(\mu_{k}/\left(\mu_{k}+v_{k}\right)\right)\right)}$$

Here $\left(\mu_{k},v_{k},\pi_{k}\right)$ is an intuitionistic fuzzy number used to assess the *k*^th^ expert. The $\omega_{k}$ means the weight value of *k*^th^ expert.

In the fourth step, the experts’ pairwise comparisons on the criteria and subcriteria are aggregated using the IFWA aggregation operator as in Equations 7 and 8.


(7) $$r_{ij}=IFWA_{\omega }=\left(r_{ij}^{\left(1\right)},r_{ij}^{\left(2\right)},\ldots .,r_{ij}^{\left(t\right)}\right)=\omega_{1}r_{ij}^{\left(1\right)}⨁\omega_{2}r_{ij}^{\left(2\right)}⨁\ldots ⨁\omega_{t}r_{ij}^{\left(t\right)}$$

(8) $$IFWA_{\omega }=\left(1-\prod_{k=1}^{t}{\left(1-\mu_{ij}^{\left(k\right)}\right)}^{\omega_{k}},\prod_{k=1}^{t}{\left(v_{ij}^{\left(k\right)}\right)}^{\omega_{k}},\prod_{k=1}^{t}{\left(1-\mu_{ij}^{\left(k\right)}\right)}^{\omega_{k}}-\prod_{k=1}^{t}{\left(v_{ij}^{\left(k\right)}\right)}^{\omega_{k}}\right)$$

Here, $R^{\left(k\right)}={\left(r_{ij}^{\left(k\right)}\right)}_{mxn}$ is an intuitionistic fuzzy decision matrix of the *k*^th^ expert and $r_{ij}=\left(\mu_{ij},v_{ij},\pi_{ij}\right)$.

In the fifth step, the consistency ratio (CR) for the aggregated intuitionistic fuzzy decision matrix has been computed. The traditional CR computation procedure of Saaty [49-53] is mainly suggested for all types of fuzzy set extensions.

In the sixth step, the intuitionistic fuzzy weights of the aggregated intuitionistic fuzzy decision matrix are calculated using Equations 9 and 10.


(9) $${\overset{̿}{w}}_{i}=-\frac{1}{nln2}\left(\mu_{i}ln\mu_{i}+v_{i}lnv_{i}-\left(1-\pi_{i}\right)\ln\left(1-\pi_{i}\right)-\pi_{i}ln2\right)$$

(10) $$\;\;w_{i}=\frac{1-{\overset{̿}{w}}_{i}}{n-\sum_{i=1}^{n}{\overset{̿}{w}}_{i}}$$

Ranks of the criteria and subcriteria are obtained in the seventh and last step of the IF-AHP algorithm. It must be noted that if the values are nonnormalized, they must be normalized before finding the final optimal values.

### IF-DEMATEL Algorithm

After the steps of the IF-AHP algorithm are given above, the application of the IF-DEMATEL algorithm, which will investigate the dependency relationship between the criteria in the second part, has been started. A more straightforward understanding of the notation here depends on comprehending the intuitionistic fuzzy set notation presented in the previous section. The steps of the IF-DEMATEL algorithm are as follows.

As performed at the beginning of IF-AHP, the first step involves determining the evaluation criteria and subcriteria inside the problem.

The second step of IF-DEMATEL is to build a direct relation matrix. Evaluations of the expert members of the team are made by consensus. Here, a 2-tuple intuitionistic linguistic scale is preferred as follows: “null influence” (0.1, 0.9), “low influence” (0.35, 0.6), “medium influence” (0.5, 0.45), “high influence” (0.75, 0.2), and “very high influence” (0.9, 0.1).

In the third step, the equivalent fuzzy subset’s related membership degree is computed by Anzilli and Facchinetti’s procedure [44], as detailed in the IF-AHP algorithm section. By this procedure, the intuitionistic fuzzy sets are converted to a corresponding standard fuzzy subset; thus, the “initial direct relation matrix” in standard fuzzy subsets is built.

In the fourth step, the standard fuzzy subset values are defuzzified; thus, a crisp initial direct relation matrix is built.

The fifth step is on normalizing the direct-relation matrix, which is constructed in the previous step (Step 4). The normalized direct-relation matrix (*G*) is computed following the traditional crisp data-based DEMATEL steps as in Equations 11-13.


(11) $$G=g^{-1}X$$

(12) $$g=max\left(\underset{1\leq i\leq n}{\max}\sum_{j=1}^{n}x_{ij},\underset{1\leq j\leq n}{\max}\sum_{i=1}^{n}x_{ij}\right)$$

(13) $$X={\left(x_{ij}\right)}_{nxn}={\left(\frac{\sum_{K=1}^{h}w_{k}x_{ij}^{k}}{\sum_{K=1}^{h}w_{k}}\right)}_{nxn}$$

where,$w_{k}$ demonstrates the weight of expert-*k*. The *X* matrix is the aggregated direct-relation matrix.

The sixth step is to form the total relation matrix (*T*) by Equation 14:


(14) $$T=G{\left(I-G\right)}^{-1}$$

where, *I* is the identity matrix. In this step, the net cause and effects are identified. Equations 15 and 16 are the computational formulas of prominence $\left(D+R^{T}\right)$ and relation $\left(D-R^{T}\right)$ vectors.


(15) $$D={\left(\sum_{j=1}^{n}t_{ij}\right)}_{nx1}={\left(t_{i}\right)}_{nx1}$$

(16) $$R={\left(\sum_{i=1}^{n}t_{ij}\right)}_{1xn}={\left(t_{j}\right)}_{1xn}$$

The seventh and final step of the IF-DEMATEL algorithm is finalized by drawing the $\left(D+R^{T}\right)$-$\left(D-R^{T}\right)$ digraph map.

### The CoCoSo Method

CoCoSo was proposed as a miscellaneous mix of simple additive weighting (SAW), weighted aggregated sum product assessment, and multiplicative exponential weighting methods [35,37,54-56]. Its algorithm includes several steps, as given below [37].

The first step is to generate an initial decision matrix. It is referred to in Equation 17. Here i refers to the candidate classifying algorithms. On the other side, j refers to the decision criteria and subcriteria regarding the selection of classification algorithms supporting effective AT allocation, fostering independent living, and reducing the economic and social burden faced by patients with PD mentioned in the IF-AHP and IF-DEMATEL sections.


(17) $$A=\left[a_{ij}\right]$$

In the second step, the initial decision matrix is normalized following Equations 18 and 19.


(18) $$r_{ij}=\frac{a_{ij}-\min_{i}a_{ij}}{\max_{i}a_{ij}-\min_{i}a_{ij}}\;for\;\;benefit\;\;criteria$$

(19) $$r_{ij}=\frac{\max_{i}a_{ij}-a_{ij}}{\max_{i}a_{ij}-\min_{i}a_{ij}}\;for\;\;\;cost\;\;criteria$$

The third step of CoCoSo is to calculate the sum of weighted comparability ${(S}_{i})$ value and power-weighted comparability sequences ${(P}_{i})$ for each alternative classifying algorithm via Equations 20 and 21.


(20) $$S_{i}=\sum_{j=1}^{n}w_{j}r_{ij}$$

(21) $$P_{i}=\sum_{j=1}^{n}{r_{ij}}^{w_{j}}$$

In the fourth step, 3 different aggregated appraisal scores $\left(M_{ia},M_{ib},M_{ic}\right)$ are introduced to compute the weights of each alternative classifying algorithm via Equations 22-24.


(22) $$M_{ia}=\frac{P_{i}+S_{i}}{\sum_{i=1}^{m}\left(P_{i}+S_{i}\right)}$$

(23) $$M_{ib}=\frac{S_{i}}{\underset{i}{\min}S_{i}}+\frac{P_{i}}{\underset{i}{\min}P_{i}}$$

(24) $$M_{ic}=\frac{\lambda \left(S_{i}\right)+(1-\lambda )\left(P_{i}\right)}{\lambda \underset{i}{\max}S_{i}+(1-\lambda )\underset{i}{\max}P_{i}}$$

The fifth and last stage of CoCoSo focuses on finding the ranking of each alternative classifying algorithm considering the descending order $M_{i}$ scores via Equation 25.


(25) $$M_{i}=\sqrt[{3}]{\left(M_{ia}M_{ib}M_{ic}\right)}+\frac{1}{3}\left(M_{ia}+M_{ib}+M_{ic}\right)$$

### Ethical Considerations

According to UK regulations (UK Research and Innovation, 2024 [57]), ethical approval was not required for this study as it did not involve human participants.

## Results

### Overview

The proposed approach was implemented using the PD data derived from the iPhone app called mPower [58]. In detail, 74 adopters and 307 nonadopters were enrolled in this project. Each participant was required to undertake 4 activity types supported by the app: voice, tipping, walking, and typing. In all, 3 classification algorithms—naive Bayes, J48 decision tree, and lazy instance-based *k*-NN (IBK)—were candidates to predict AT adoption in this context as stipulated in [59]. However, this study only focused on the performance indicators and did not consider other aspects of the app context, including usability, design, scalability, and flexibility. Such factors may limit the implementation of high-accurate algorithms in the clinical scenario, thereby limiting the exploitation of the app benefits. In the meantime, not assessing these aspects may trigger cost overruns for the health care system and have potential detrimental effects on patients with PD. This has also represented a challenge for data analytics experts, who must design classifiers highly adaptable to the environment and the changing dynamics of the health care sector. The following subsections will describe how the multimethod MCDM framework has been applied to indicate which algorithm should be selected to effectively discriminate the potential mPower adopters and nonadopters while considering the practical clinical scenario.

### The Decision-Making Group

A pertinent decision-making team from the REMIND project Consortium [60] was needed to pinpoint the criteria/subcriteria importance and the interrelations in the decision model that support the technology adoption in patients with PD. In particular, the team participants are expected to: (1) define the decision factors integrating the classifier selection model; (2) undertake the necessary pairwise comparisons to obtain the relative priorities of the factors in the presence of uncertainty, vagueness, and hesitancy; (3) perform judgments to assess the significant cause-effect interrelations affecting the deployment of classifiers in the wild; and (4) contribute to the design of recommendations for improving the suitability/transferability of the classifiers concerning the real health care scenario. This intervention was guided by 1 researcher coauthoring this paper (MO-B) and had the participation of 8 experts from different disciplines whose profiles are described in Table 1. All these experts have been directly involved in designing assistive technology solutions for patients with PD and consequently have extensive knowledge of the decision-making scenario.

**Table 1.** Profile of experts enrolled in the classifier selection process.

Expert	Profession	Areas of expertise	Experience (years)	Current position
E_1_	Biomedical engineer	Technology adoption modeling – mobile-based reminding solutions	30	Managing director
E_2_	Informatics engineer	Artificial intelligence – pervasive and mobile computing	>10	Researcher
E_3_	Biomedical engineer	Ambient assisted living – pervasive and mobile computing	>10	Senior lecturer
E_4_	Computer science engineer	Pervasive and mobile computing	>10	Senior lecturer
E_5_	Electrical engineer	Image processing – artificial intelligence models	>10	Professor
E_6_	Computer science engineer	Health innovation – health technology	>10	Professor
E_7_	Informatics engineer	Artificial intelligence – pervasive and mobile computing	>10	Data scientist

In this application, the project leader designed the classifier selection model by including the decision criteria/subcriteria and candidate algorithms elucidated with the aid of the decision-making group, the health care providers, the pertinent scientific literature, and the applicable health guidelines. Moreover, he trained the decision-makers to make correct judgments using IF-AHP and IF-DEMATEL techniques. A virtual data-collection tool was prepared and later used by the participants, who finished all the necessary comparisons during a 1-hour session. This process raised awareness in the decision-making group of the factors AT developers should take into account when designing and deploying the classifiers in the actual health care context. Usually, the data experts are inclined to enhance the performance of these algorithms without considering how they should be implemented in the wild. Therefore, including all these aspects will empower AT developers to comprehend the health care scenario and define action lines transforming classifiers in a feasible technology adoption support in people with PD.

### The Classifier Selection Network

**Figure 2.**
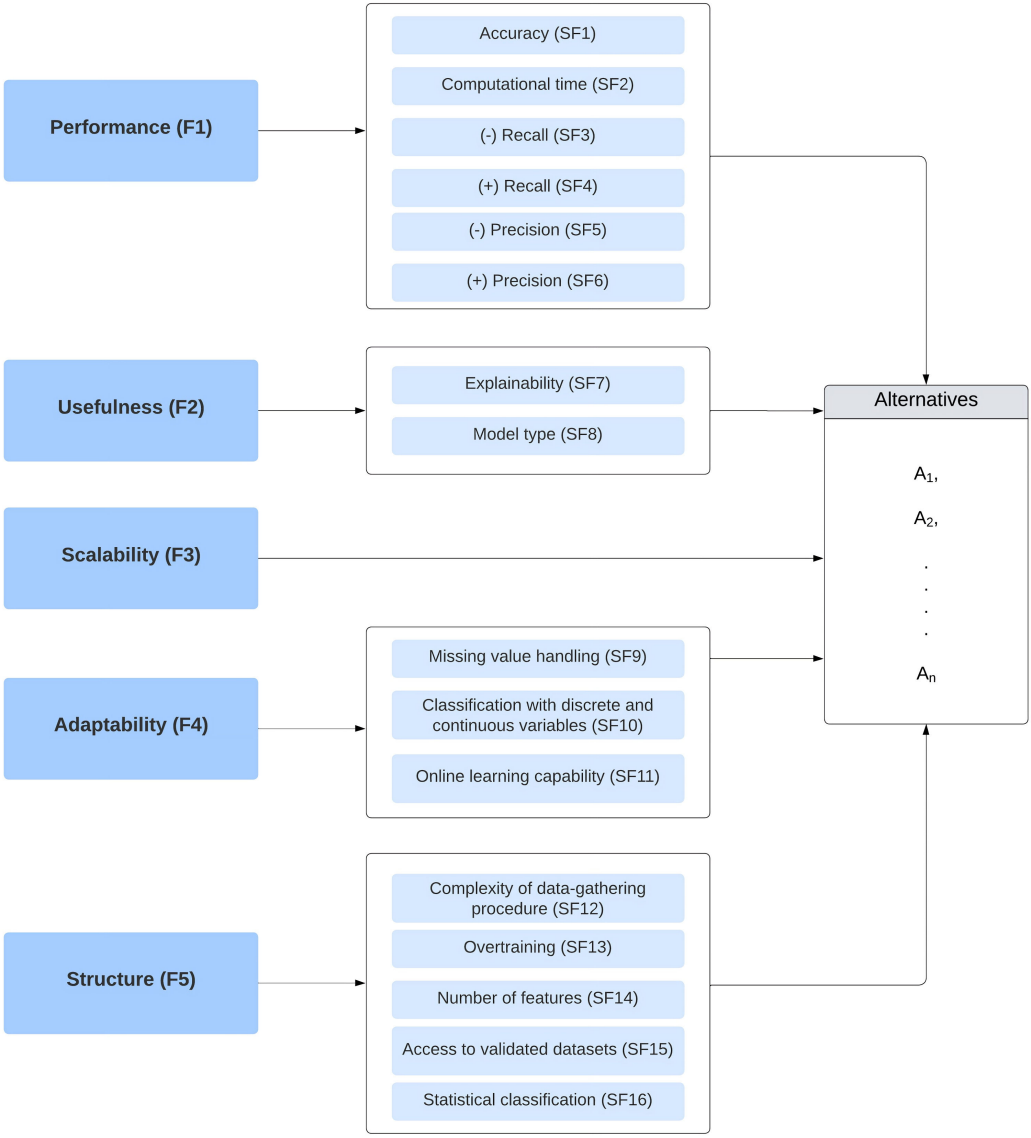
The classifier selection network for underpinning technology adoption in people with Parkinson disease.

**Figure 3.**
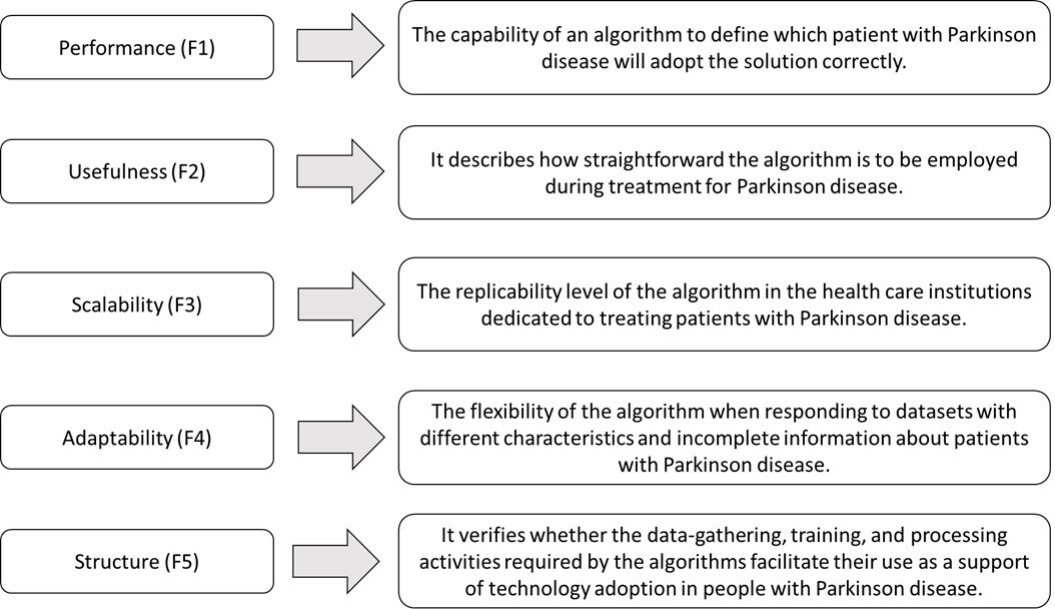
Description of classifier selection factors included in the network model.

The classifier selection network designed for underpinning technology adoption in patients with PD was studied together with the decision-making group to determine if it was suitable, coherent, reasonable, and deployable in the real world. The ensuing model (Figure 2) is composed of 5 factors, 16 subfactors, and 3 algorithms. Figure 3 outlines each element complemented by supplementary descriptions of the subfactors incorporated into the network.

The decision factors have been subdivided into more detailed aspects to provide a more complete panorama of the suitability of classifiers. At the same time, there is a need to pinpoint improvements that can be translated into more applicable algorithms. For instance, erformance (F1) has 6 subelements: accuracy (SF1), computational time (SF2), (−) recall (SF3), (+) recall (SF4), (−) precision (SF5), and (+) precision (SF6). Accuracy is the number of correct classifications (adopter/nonadopter) divided by the total number of classifications. On the other hand, computational time refers to the velocity at which the classifier predicts whether the patient with PD can adopt the technology effectively. (−) Recall defines how well the classifier identifies the patients who cannot assume the assistive solution, which avoids potential adverse effects on their self-esteem and life expectancy. Meanwhile, (+) recall measures how well the algorithm discriminates against patients with PD who can suitably assume the technology, making it possible to upgrade their life quality while decreasing delayed intervention. On a different tack, (−) precision (SF5) measures the relation between the true negative cases and the predicted negative cases, while (+) precision (SF6) denotes the same ratio but considers positive cases.

Conversely, sefulness has been split into explainability (SF7) and model type (SF8). The first subcategory denotes whether the doctor/nurse can identify and interpret the technology adoption decision recommended by the algorithm for a specific patient with PD. Likewise, model type establishes whether the algorithm is black-box or white-box.

In the adaptability cluster, 3 decision elements are enlisted: missing value handling (SF9), classification with discrete and continuous variables (SF10), and online learning capability (SF11). Health care datasets are often characterized by presenting incomplete information and/or registration errors regarding critical patient data [61,62]; this is why it is necessary to determine if the classifier can deal with this problem without further affecting their functionality. In addition, it is essential to define if the classifier can cope with discrete and continuous patient metrics, as evidenced in all the big data systems supporting Parkinson-related health care services [63]. Furthermore, it is expected to have classifiers that can be adapted according to the dynamic context of PD and the health care scenario. In other words, the algorithm should evolve by including significant emerging features responding to the context changes.

Ultimately, the structure criterion comprises 5 aspects: complexity of data-gathering procedure (SF12), overtraining (SF13), number of features (SF14), access to validated datasets (SF15), and statistical classification (SF16). Complexity of data-gathering procedure establishes if the algorithm imports the dataset from a low number of self-administered questions or retrospectively. On a different note, some classifiers experience overtraining difficulties, which indicates an apparent performance improvement but entails a worse generalization of the test data. This problem has been extensively reported in the ML literature and can only be noticed once real technology adoption is adequately detected [64,65]. In the implementation phase, it is preferable to use classifiers requiring few features to decide whether the patient with PD can adopt a particular solution; otherwise, the procedure supporting this decision will be time-consuming and less feasible in the real world. It is additionally expected that classifiers have access to validated data as it allows them to avoid corrupted data that could possibly affect the performance of classifiers. Ultimately, statistical classification algorithms enable decision-makers to define which factors are more significant in technology adoption for people with PD. They provide coefficients whose dimensionality and direction denote if each variable substantially/hardly increases or decreases the adoption likelihood.

### Intuitionistic Fuzzy Relative Priorities of Criteria and Subcriteria: The IF-AHP Application

The IF-AHP technique was used to compute the relative weights of criteria and subcriteria in the classifier selection network. In this regard, a virtual survey was designed to collate the comparisons based on the assessment scale suggested in the Intuitionistic Fuzzy Analytic Hierarchy Process section. Following this, coefficients were assigned to the decision-makers using the scheme proposed by Boran et al [48]. In this case, the decision-maker (experts; E_k_) with the greatest relevance was E_1_ (0.2857), taking into account their comprehensive knowledge and background in the design and application of IT solutions for health care (Table 2). Afterward, the pairwise comparisons derived from the Es were aggregated by the IFWA operator (Equations 7 and 8). An example of this stage is presented in Table 3 for the flexibility subcriteria. This matrix was then normalized by Equations 9 and 10, as evidenced in Table 4. Table 5 depicts the resulting local weight and overall weights of factors and subfactors. The CR of each cluster was computed using Saaty’s approach [49,50]: factors (0.04), performance (0.002), usefulness (0), adaptability (0.06), and structure (0.01).

**Table 2.** Priorities of decision-makers.

Expert	Intuitionistic fuzzy number			Priority
E_1_	(0.9, 0.05, 0.05)			0.2857
E_2_	(0.75, 0.2, 0.05)			0.2380
E_3_	(0.75, 0.2, 0.05)			0.2380
E_4_	(0.75, 0.2, 0.05)			0.2380
E_5_	(0.75, 0.2, 0.05)			0.2380

**Table 3.** Aggregated intuitionistic fuzzy matrix for flexibility subcriteria.

	SF9	SF10	SF11
SF9	[0.020, 0.180, 0.800]	[0.099, 0.176, 0.724]	[0.099, 0.176, 0.724]
SF10	[0.099, 0.176, 0.659]	[0.020, 0.180, 0.800]	[0.074, 0.159, 0.766]
SF11	[0.099, 0.176, 0.724]	[0.074, 0.159, 0.766]	[0.020, 0.180, 0.800]

**Table 4.** The normalized priorities of flexibility subcriteria.

	Intuitionistic fuzzy weight			Nonfuzzy weight	Overall weight
SF9	0.073	0.177	0.749	0.292	0.069
SF10	0.065	0.172	0.742	0.267	0.063
SF11	0.065	0.172	0.763	0.273	0.065
Total	—^a^	—	—	0.833	0.198

a Not applicable.

**Table 5.** The local weight and overall weight of factors and subfactors in the classifier selection model.

Criteria/subcriteria	Local weight	Overall weight
**Performance (F1)**	—^a^	0.187
Accuracy (SF1)	0.180	0.034
Computational time (SF2)	0.193	0.036
(–) Recall (SF3)	0.157	0.029
(+) Recall (SF4)	0.160	0.030
(–) Precision (SF5)	0.156	0.029
(+) Precision (SF6)	0.154	0.029
**Usefulness (F2)**	—	0.199
Explainability (SF7)	0.500	0.100
Model type (SF8)	0.500	0.100
**Scalability (F3)**	—	0.198
**Adaptability (F4)**	—	0.202
Missing value handling (SF9)	0.351	0.069
Classification with discrete and continuous variables (SF10)	0.321	0.063
Online learning capability (SF11)	0.328	0.065
**Structure (F5)**	—	0.214
Complexity of data-gathering procedure (SF12)	0.177	0.038
Overtraining (SF13)	0.207	0.044
Number of features (SF14)	0.212	0.045
Access to validated datasets (SF15)	0.223	0.048
Statistical classification (SF16)	0.181	0.039

a Not applicable.

### Intuitionistic Fuzzy Interdependence and Feedback: The IF-DEMATEL Approach

The next step of this approach was to study the interrelations among the classifier selection factors/subfactors to identify interventions in the long-term classifier development and technology adoption processes. The 2-tuple intuitionistic linguistic scale for assessing the influence between the factors/subfactors (Intuitionistic Fuzzy Decision-Making Trial and Evaluation Laboratory section) was first explained to the experts. The decision-makers then made the judgments using an easy-to-manage data-collection tool during a 3-hour session. Table 6 presents the initial intuitionistic fuzzy direct-relation matrix derived from E_3_ concerning the adaptability subfactors. As a next step, the IFS were crisped by a 2-step procedure. First, the IFS were transformed into their respective subsets using the equation $\mu \left(x\right)=\frac{1}{2}(1+\mu_{I}\left(x\right)-v_{I}\left(x\right))$ (Table 7). A crisp function was later applied to convert the intuitionistic fuzzy subset into a crisp value. In this respect, a crisp initial direct relation matrix is generated when allocating the values in Table 7 to the triangular fuzzy vector <0, 4, 4> (Table 8). We then aggregated the defuzzified values of all experts using the simple mean (Table 9). The next stage was to compute the normalized direct-relation matrix (*G*) by applying Equations 11-13 (Table 10). The total relation matrix *T* (Table 11) was then derived by using Equation 14. Ultimately, Table 12 presents the prominence (D+R) and relation (D–R) values resulting from Equations 15 and 16 to define which factors or subfactors can be grouped into the driving and effect categories. The developers should be focused on the main drivers to make the classifiers more adaptable to the health care scenario and the technology adoption requirements.

**Table 6.** Initial intuitionistic fuzzy direct-relation matrix – E_3_ (adaptability subfactors).

	SF9		SF10		SF11	
SF9	0	0	0.75	0.2	0.1	0.9
SF10	0.75	0.2	0	0	0.1	0.9
SF11	0.5	0.45	0.5	0.45	0	0

**Table 7.** Initial intuitionistic fuzzy direct-relation matrix – E_3_ in subsets (adaptability subfactors).

	SF9	SF10	SF11
SF9	0	0.78	0.1
SF10	0.78	0	0.1
SF11	0.53	0.53	0

**Table 8.** Crisp direct-relation matrix for adaptability subcriteria – E_3._

	SF9	SF10	SF11
SF9	0	3.1	0.4
SF10	3.1	0	0.4
SF11	2.1	2.1	0

**Table 9.** Aggregated direct-influence matrix for adaptability subcriteria.

	SF9	SF10	SF11
SF9	0	2.175	1.912
SF10	2.65	0	2.225
SF11	2.662	2.662	0

**Table 10.** Normalized aggregated direct-influence matrix for adaptability subcriteria.

	SF9	SF10	SF11
SF9	0	0.408	0.359
SF10	0.498	0	0.418
SF11	0.5	0.5	0

**Table 11.** Total influence matrix for adaptability subcriteria.

	SF9	SF10	SF11	D
SF9	2.387	2.518	2.269	7.174
SF10	3.026	2.513	2.555	8.093
SF11	3.206	3.016	2.412	8.634
R	8.619	8.047	7.235	—^a^

a Not applicable.

**Table 12.** Dispatchers and receivers in the classifier selection network.

	D+R	D–R	Category
F1	9.924	–0.793	Effect
SF1	9.403	0.969	Driver
SF2	7.948	–1.054	Effect
SF3	8.397	–0.604	Effect
SF4	8.947	0.168	Driver
SF5	8.838	0.395	Driver
SF6	8.768	0.127	Driver
F2	9.879	–1.026	Effect
SF7	33.545	–1.000	Effect
SF8	33.545	1.000	Driver
F3	9.882	–0.118	Effect
F4	10.481	1.234	Driver
SF9	15.794	–1.445	Effect
SF10	16.140	0.047	Driver
SF11	15.868	1.399	Driver
F5	10.423	0.703	Driver
SF12	18.864	0.620	Driver
SF13	16.530	–2.369	Effect
SF14	18.861	1.024	Driver
SF15	18.733	1.115	Driver
SF16	18.385	–0.390	Effect

**Figure 4.**
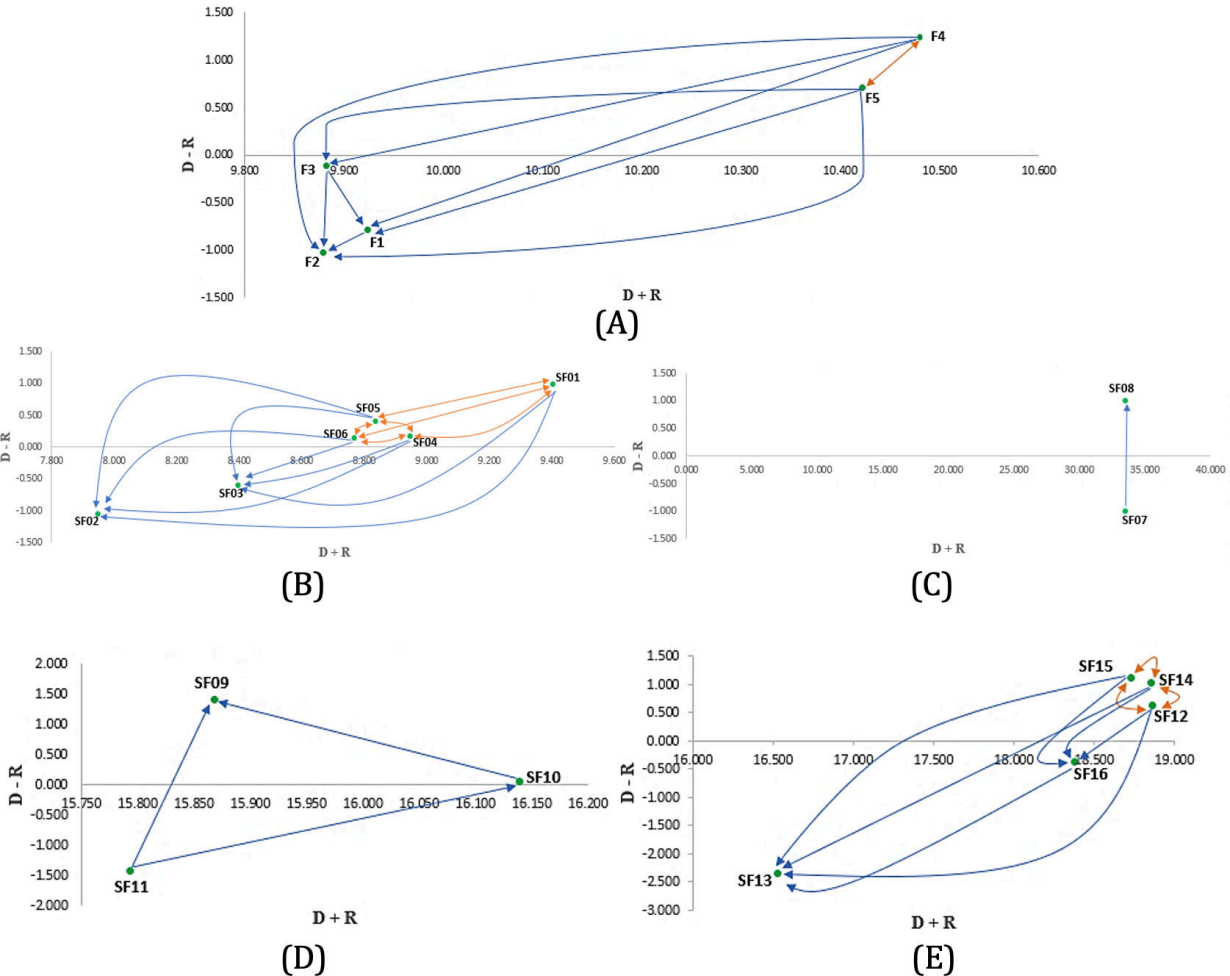
Impact-digraph maps for (**A**) factors, (**B**) performance, (**C**) usefulness, (**D**) adaptability, and (**E**) structure.

$\left(D+R^{T}\right)-\left(D-R^{T}\right)$ digraph maps (Figure 4A–4E) were also built to examine the interrelations among the factors/subfactors underpinned by the computation of reference values elucidating the significant influences. The developers must carefully intervene in these influences in conjunction with the health care staff to ensure high-deployable classification algorithms.

### Ranking of Classifiers: The CoCoSo Implementation

This section outlines the CoCoSo application, whose main objective is two-fold: (1) to derive the transferability index ($M_{i}$ score) helping to rank the classifier alternatives, namely, lazy IBK – *k-*NN (A1), naive Bayes (A2), and J48 decision tree (A3), that may support technology adoption in people with PD; and (2) to detect those characteristics that should be improved in each algorithm to better support this decision in the wild. The CoCoSo implementation was initiated by setting a metric per each classifier selection criterion/subcriterion. The list of indicators and their formula are presented in Table 13. These indexes were established considering the pertinent scientific evidence and the health care context associated with PD. The values of each decision element and classifier were included in the initial decision matrix *A* (Tables 14 and 15). This arrangement (Equation 17) also incorporates the overall weights *w* computed by using the IF-AHP technique (for more information, see the section titled The CoCoSo Method).

**Table 13.** List of metrics and their calculation method.

Classifier selection criterion/subcriterion	Metric	Formula
Accuracy (SF1)	Average accuracy	$\sum_{i=1}^{n}\left(\frac{TNC+TPC}{TPC+FPC+FNC+TNC}\right)*\frac{100}{n}$ TNC: true negative cases TPC: true positive cases FPC: false positive cases FNC: false negative cases n: number of iterations
Computational time (SF2)	Average time complexity	$\sum_{i=1}^{n}\frac{IT_{i}}{n}$ n: number of iterations IT_i_: iteration time per instance i
(–) Recall (SF3)	Average negative recall	$\sum_{i=1}^{n}\left(\frac{TNC}{FPC+TNC}\right)*\frac{1}{n}$ TNC: true negative cases FPC: false positive cases n: number of iterations
(+) Recall (SF4)	Average positive recall	$\sum_{i=1}^{n}\left(\frac{TPC}{TPC+FNC}\right)*\frac{1}{n}$ TNC: true positive cases FNC: false negative cases n: number of iterations
(–) Precision (SF5)	Negative positive precision	$\sum_{i=1}^{n}\left(\frac{TNC}{TNC+FNC}\right)*\frac{1}{n}$ TNC: true negative cases FPC: false negative cases n: number of iterations
(+) Precision (SF6)	Average positive precision	$\sum_{i=1}^{n}\left(\frac{TPC}{TPC+FPC}\right)*\frac{1}{n}$ TNC: true positive cases FPC: false positive cases n: number of iterations
Explainability (SF7)	Interpretability	If the algorithm is simple to interpret by a doctor and/or nurse (2), otherwise (1)
Model type (SF8)	Model category	If the model is a black box (2), white box (1)
Scalability (F3)	Cost classification	If the learning cost overpasses €927 (US $1018) (1), otherwise (2)
Missing value handling (SF9)	Missing value management	If the algorithm supports datasets with missing values (2), otherwise (1)
Classification with discrete and continuous variables (SF10)	Data type	If the classification model supports continuous and discrete data (2), otherwise (1)
Online learning capability (SF11)	Online learning	If the classifier is trained through online learning (2), otherwise (1)
Complexity of data-gathering procedure (SF12)	Data-gathering management	If a self-administered survey is used for collecting the feature set (2), otherwise (1)
Overtraining (SF13)	Overtraining	If the algorithm has overtraining problems (2), otherwise (1)
Number of features (SF14)	Number of features	Number of the input variables requested by the algorithm to perform the technology adoption prediction
Access to validated datasets (SF15)	Classifier validation	If the algorithm can be verified with validated datasets (2), otherwise (1)
Statistical classification (SF16)	Statistical capability	If the model is statistical (2), otherwise (1)

**Table 14.** Initial decision matrix A – SF01 to F4.

Algorithm	SF1	SF2	SF3	SF4	SF5	SF6	SF7	SF8	F3
A1	73.36	0.00011	0.72	0.74	0.73	0.73	2	2	1
A2	69.05	0.0000	0.74	0.63	0.71	0.67	2	2	1
A3	76.98	0.0017	0.83	0.71	0.81	0.74	2	1	2
Overall weight	0.034	0.036	0.029	0.030	0.029	0.029	0.100	0.100	0.198

**Table 15.** Initial decision matrix A – SF9 to SF16.

	SF09	SF10	SF11	SF12	SF13	SF14	SF15	SF16
A1	2	2	1	2	1	5	2	1
A2	2	2	2	2	1	5	2	2
A3	2	2	1	2	1	5	2	2
Overall weight	0.069	0.063	0.065	0.038	0.044	0.045	0.048	0.039

The initial matrix *A* was then normalized following Equations 18 and 19. After this, we computed the ${(S}_{i})$ and ${(P}_{i})$ for each classifier (Table 16). The next step involved estimating the aggregated appraisal scores $\left(M_{ia},M_{ib},M_{ic}\right)$ via Equations 20-22 with λ=0.5 (Table 16). Finally, the transferability index $M_{i}$ score (Equation 23) was derived for each classifier (Table 16).

**Table 16.** Aggregated appraisal scores and ranking of classifiers.

	*S_i_*	*P_i_*	*M_ia_*	*M_ib_*	*M_ic_*	*M_i_*	Ranking
A1	0.5213	11.929	0.3126	2.0892	0.7841	1.8620	2
A2	0.5523	10.952	0.2888	3	0.7246	1.7796	3
A3	0.8868	14.990	0.3986	3.0700	1.0000	2.5592	1

### Validation Study: Contrasting CoCoSo Results With TOPSIS and SAW

**Figure 5.**
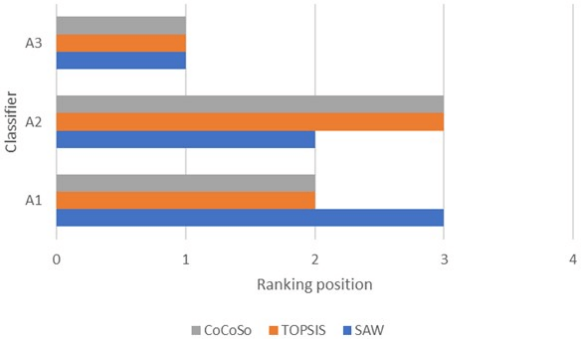
Ranking of classifiers according to CoCoSo, SAW, and TOPSIS. CoCoSo: combined compromise solution; SAW: simple additive weighting; TOPSIS: technique for order of preference by similarity to ideal solution.

Even though we have suggested a robust strategic methodology combining 3 MCDM techniques with the intuitionistic fuzzy logic, it is always necessary to validate its accuracy compared to well-known methods. In this sense, we contrasted the scoring technique used in the last phase (CoCoSo) with SAW and TOPSIS. The resulting rankings in each method are shown in Figure 5. Upon analyzing this graph, no changes were observed in A3, which was the most suitable classifier in all 3 approaches. There is a slight variation in the SAW ranking of A1 and A2 compared to the findings derived from TOPSIS and CoCoSo. This is expected, considering the differences in each method’s normalization and scoring procedures. These results then underpin the accuracy and applicability of the suggested methodology.

**Figure 6.**
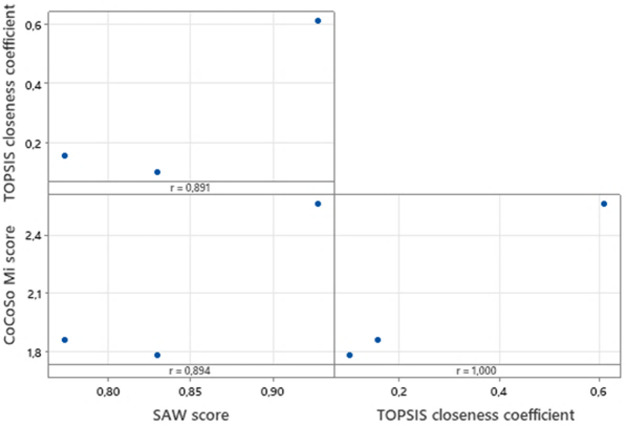
Pearson correlation tests between transferability indexes of TOPSIS, SAW, and CoCoSo. CoCoSo: combined compromise solution; SAW: simple additive weighting; TOPSIS: technique for order of preference by similarity to ideal solution.

Furthermore, the Pearson correlation tests (Figure 6) were conducted considering the transferability indexes derived from each method. The scores are highly correlated (*r*>0.8), especially when comparing CoCoSo and TOPSIS (*r*=1). This procedure strengthens the graphical validation performed in Figure 5.

## Discussion

### Principal Results, Limitations, and Comparison With Previous Work

#### The Importance of Classifier Selection Criteria and Subcriteria

Considering the IF-AHP results, structure (F5) was identified as the factor with the highest relative priority. However, there was no significant difference between this factor and the other factors involved in the selection model (F5 vs F4=0.012; F5 vs F2=0.015; F5 vs F3=0.016; F5 vs F1=0.027). This demonstrates that all these factors should be simultaneously considered when selecting classifiers supporting technology adoption in patients with PD. Specifically, structure is identified as an essential factor in the selection process, given the need to accelerate the deployment of the classifiers in the actual technology scenario. Algorithms with overtraining problems, complex data-collection procedures, a high number of input features, no access to validated datasets, and no statistical modeling may enlarge the learning curve in health care staff and trigger a high rate of incorrect classifications. This finding is confirmed by Badillo et al [66], who identified that an inadequate or deficient model structure could affect the key variable predictions, which, in the case of the technology adoption processes, may signify fair discrimination between adopters and nonadopters of assistive technologies [67]. Therefore, efforts should be directed at improving the structural characteristics of the classifiers to optimize the technology adoption process within the health care scenario. Thereby, the rate of rejection and abandonment of technology can be reduced while improving the quality of life of patients with PD and their families.

It is also essential to analyze the ranking of classifier selection subfactors to derive more focused interventions. In this case, the first 2 (explainability and model type) correspond to the usefulness domain. The importance of these subcriteria lies in the fact that the selected classifiers should be easy to manage and interpret for nonexpert users such as doctors and support staff. On the contrary, there will be some resistance to change, lack of interest, extended learning time, and subsequent delays in technology adoption. These findings confirm what Miotto et al [68] reported regarding the importance of the model’s explainability and the interpretability of the results as critical aspects in developing reliable technology assistance in patients with PD.

The following 3 subfactors in the ranking are missing value handling, online learning capability, and classification with discrete and continuous variables, which belong to the adaptability factor, the second most crucial factor in the selection model. Missing value handling is one of the most common and intrinsic problems in handling large volumes of health care data [69,70]. There are several reasons for missing data, including poor adherence to data handling procedures and policies and unsuitable reporting mechanisms. Consequently, the technology adoption classifiers must be able to identify and impute the lost data adequately to avoid biases or false results that can lead medical and support staff to make wrong decisions when allocating a specific solution. This poses a challenge for developing studies focused on improving the handling of missing data. Removing values, assigning default values, or blaming the data have been some of the reported missing data approaches [71-73]. For instance, Prince et al [74] demonstrated the ability to predict PD in the presence of missing values by dividing the dataset into 2 subgroups comprising people with missing and complete source data. On the other hand, the online learning capability implies that technology adoption algorithms must continuously evolve by incorporating new features according to advances in the diagnosis and management of PD, both at the clinical and home care levels, which validate the findings of Ortiz-Barrios et al [5]. One of the strategies that can be adopted to improve the learning capacity of the classifier is the one proposed by Sigcha et al [75], in which a pretrained transfer learning model was designed to enhance the technology adoption in natural environments. Finally, the importance of the classification with discrete and continuous variables lies in the ability of classifiers to receive data of a different nature in the context of PD. For example, Harimoorthy and Thangavelu [76] mentioned that one of the main criteria in PD-related prediction models is the collection of patients’ voice characteristics, whose nature may be discrete or continuous.

Ultimately, consistency rates were computed for the aggregated intuitionistic fuzzy decision matrixes based on Saaty [49,54]. The results showed that all matrices were consistent (CR<0.1), demonstrating the decision-making process’s robustness regarding the estimated priorities of factors and subfactors. These outcomes are supported by an adequate selection of experts complemented with training and guidance during the evaluation process. In addition, it is important to remark on the importance of using easy-to-manage surveys and the shorter version of Saaty’s scale to reduce assessment bias [77-80]. The sound effects of these practices are also evident in large matrices (n≥5; performance and structure subcriteria) where the CR was equal to or less than 0.01.

#### Interdependence Assessment in the Classifier Selection Network

IF-DEMATEL shows that adaptability (F4) and structure (F5) are the dispatchers while performance (F1), usefulness (F2), and scalability (F3) belong to the effect group. Therefore, developers, personnel, and physicians must establish intervention actions focused on the driver factors to support the technology adoption process in patients with PD in the long term. In addition, structure and adaptability present the highest prominence value, being the primary influencers in the classifier selection model and then become priority factors that need to be carefully considered in ML algorithm design approaches for the PD context. These results are consistent with the findings of Sigcha et al [75], who highlighted that the architecture, the training configurations, and the learning model parameters are essential for the adequate scalability of the discrimination results. In this sense, a flexible model architecture and the documentation of all the model construction stages are strongly recommended for making the technology adoption process more efficient. Therefore, classifiers with these characteristics must have a high probability of being selected to support this process in the health care scenario. These conclusions are also underpinned by the presence of a feedback relationship between the aforementioned elements (Figure 4A), where it is evident how the data collection, training, and processing highly restrict the adaptability of the classifier to the PD context.

In addition, influence maps (Figure 4B–E) were prepared to show the inner interactions in each cluster and establish action courses for improving the suitability of PD technology adoption classifiers. Regarding the performance criterion (Figure 4B), the threshold value was defined as $\theta =\frac{19.20}{6^{2}}=0.53$, which helped to elucidate the significant dependencies. In conclusion, accuracy (SF1), (+) recall (SF4), (+) precision (SF5), and (–) precision (SF6) are the effect generators or dispatchers, while computational time (SF2) and (−) recall (SF3) are the receivers. It is also essential to emphasize the feedback relationships (orange arrows): SF1-SF5, SF1-SF6, SF1-SF4, SF5-SF6, SF6-SF4, and SF4-SF5. These results confirm the findings provided by Pereira et al [81] related to the existing correlation between different performance metrics when selecting the most appropriate classifier. It is also essential to highlight the cause-effect relationship between accuracy (SF1) and computational time (SF2). This relationship is significant when evaluating the classifier’s performance due to the dilemma of obtaining shorter execution times at the expense of predictive capacity. In this regard, Ali et al [82] mentioned that in many assistive technology medical applications such as PD, the execution time and complexity of the algorithm are crucial parameters for effective deployment support, lower resistance to change, and adoption in the real health care scenario. Otherwise, the clinicians may perceive the models as work overload under constant pressure. Therefore, research should be oriented to developing technology adoption models with high predictive capacity but low processing times.

On a different note, a reference value $\theta =\frac{33.545}{2^{2}}=8.386$ was defined for the usefulness subfactors group (Figure 4C). The interrelationship map uncovers that explainability (SF7) is the receiver and the model type (SF8) is the dispatcher. A related work by Zhang et al [17] indicated that transparency and accessibility to visualization allow the development of assistive health care technologies that can be easily analyzed and rationally interpreted by the clinicians who will use these solutions in the daily PD management routine. These characteristics are satisfied by white-box classifiers (eg, decision tree – A3), which reduces the learning curve experienced by health care professionals.

Interdependencies were also detected between the adaptability subfactors (Figure 4D). In this regard, the threshold metric was estimated to be $\theta =\frac{23.901}{3^{2}}=2.656$. The map revealed that missing value handling (SF9) is the only receiver, while the classification with discrete and continuous variables (SF10) and online learning capability (SF11) are the dispatchers. Although there is some debate regarding the absolute need for discrete information [83], the PD dynamics demands models capable of working with new input variables, either continuous [84] or discrete [85], to represent better the technology adoption context related to these patients. The algorithms can be updated and effectively respond at a human-level AI, as argued by Cartuyvels et al [86]. Specifically, the continuous-discrete representations allow the model to capture PD contextual information better. Handling both types of variables helps to address the limitations that each one holds. Likewise, it is vital to count on ML models that can learn from real-time data so that they can evolve to respond to the changing scenario. Thereby, these models can discriminate between adopters/nonadopters effectively considering the dynamic of the technology acceptance features. In this respect, Hoi et al [87] postulated that learning from large-scale, nonstationary accurate data is still an open challenge for the developers who are called to make this process more efficient and scalable. This is partially explained by the fact that real datasets are frequently incomplete, thereby fostering the use of imputation methods addressing the missing values [88,89].

The interactions within the structure cluster (Figure 4E) are not less relevant. The digraph portrays that the complexity of data-gathering procedure (SF12), number of features (SF14), and access to validated datasets (SF15) are the main drivers ($\sum_{i=1}^{n}\left(\frac{TNC+TPC}{TPC+FPC+FNC+TNC}\right)*\frac{100}{n}$), whereas overtraining (SF13) and statistical classification (SF16) are the receivers. The presence of feedback interrelations is also glaring among SF12, SF14, and SF15, the reason why the classifier developers need to handle this triplet effectively. The inclusion of AI algorithms in the context of PD technology is facilitated when the classifiers require fewer inputs to make the predictions. Doctors and nurses are usually reluctant to use a decision-making aid if it is too complex to manage and does not offer a significant benefit compared to the current procedures and standards [90]. In addition, simpler data-gathering mechanisms are desired to stifle a potential lack of interest from medical staff, prediction inconsistencies, extended consultation times, and work overload [91]. These aspects need to be complemented by suitable access to validated datasets, which is essential to refine the accuracy/correctness of these models when pinpointing the patients with PD with the highest technology adoption probability. However, patients and health care institutions often need to be more confident in providing personal data for security and privacy reasons. This is a significant barrier to the implementation of personalized care; therefore, it requires the application of new stringent regulations better governing data collection, use, and storage [92].

#### Transferability Index and Improvement Areas

CoCoSo was utilized to calculate the transferability index of each classifier, derive the ranking in descending order, and detect areas of improvement. This is a major contribution of this paper, considering that most related studies only focus on the performance measurements to select the best classifier in technology adoption for patients with PD [12,13,84]. In this case, the outcomes uncovered that the most appropriate algorithm for supporting technology adoption in patients with PD is the A3 - J48 decision tree. Still, there are areas for improvement in each algorithm that diminish their suitability in the health care scenario:

- Moderate accuracy, (−) precision, (+) precision, (−) recall, and (+) recall levels: In this set of classifiers, intermediate accuracy levels were reported, which entails the need to include other predictors either single or hybrid to augment the capability of distinguishing between patients with PD who will accept the assistive solution and those who will not. In addition, the (−) recall values were found to be at a medium degree, which evidences the need for upgrading their ability to identify the patients with PD who are not suitable adopters of the solution and consequently circumvent latent adverse effects on their self-esteem and life expectation due to incorrect technology allocation. In a similar vein, (+) recall scores were categorized into the medium category, revealing the necessity of increasing their capability to quickly pinpoint patients with PD who can effectively assume the solution as part of their treatment. In addition, (−) precision and (+) precision values demonstrate moderate performance when predicting nonadoption and adoption of the showcased technology. It is hence suggested to (1) collect more data to train the algorithms better; (2) refine the model hyperparameters, including the regularization strength; (3) apply class weights in case of imbalance [93]; (4) use ensembling domain knowledge techniques [94]; and (5) implement data augmentation by transforming the existing datasets if data-gathering restrictions cannot be overcome [95].
- Low scalability: In this case, the training process of A1 and A2 overpasses €927 (US $1018); therefore, strategies to make them more attractive to health care administrators from a financial perspective are needed. Application-specific integrated chips may be a feasible alternative, considering their processing speed. In parallel, data decomposition methods could be used to reduce the processing complexity, accelerate training, and consequently diminish the cost of learning.
- Low flexibility: A1 and A3 are not trained through online learning, which hinders their potential to rapidly evolve according to the changing scenario of PD and the health care sector. If this is not solved, these algorithms will require retraining to be updated, which is costly and affects their scalability in hospitals [87]. In response, online learning algorithms should be applied to extract PD data arriving sequentially. It is possible to count on an updated classifier representing the PD context in real time. Some generic proposals have emerged to provide an alternative pathway to deal with this problem in the real world. For example, Lin et al [96] proposed a scalable quantile-based induction model to boost the Hoeffding tree, thereby making the algorithm more flexible and reducing storage and computational requirements. On a different note, Ferreira et al [97] proposed an extension of *k-*NN to make it more profitable in computational cost without compromising performance.

### Conclusions

This study uses a combination of the IF-AHP, IF-DEMATEL, and CoCoSo techniques to find the best classification algorithm for detecting prospective AT adoption among people with Parkinson disease. By adopting a knowledge-driven approach to AT adoption, the suggested methodology addresses the constraints of other accuracy-based methods by considering nontypical characteristics such as these solutions’ design, validation, and implementation phases.

The study emphasizes the critical importance of carefully considering classifier selection criteria and subcriteria when implementing technology for PD patients. The structure factor (F5) and scalability (F4) were identified as top priorities, indicating its essential role in accelerating classifier deployment in real-world scenarios. It was noted that inadequate model structure could lead to incorrect predictions. At the same time, low-scalable algorithms may represent a barrier to technology adoption in patients with PD.

Additionally, the explainability and model type subcriteria within the usefulness domain were highlighted as crucial. These factors ensure that selected classifiers are user-friendly and interpretable for nonexpert users, such as medical professionals and support staff. This helps mitigate resistance to change and delays in technology adoption. White-box algorithms were specifically emphasized for their transparency, enabling a deeper understanding of predictions and facilitating more effective interventions.

Although the study contributes to the literature in many aspects, the study has several limitations that must be highlighted. First, the findings are based on a specific dataset and context related to PD, potentially limiting their generalizability to different populations or health care settings. Additionally, the accuracy of the results heavily relies on the assumed expertise of the individuals involved in the decision-making process. The study acknowledges the challenge of missing data in health care datasets, emphasizing the need to carefully consider data quality and availability. Furthermore, the number of evaluated classification algorithms is limited to 3. Different ATs may be needed in various stages of PD. Similarly, the effects of chronic diseases other than PD on the choice of AT and the impact of these conditions on the selection of the classification algorithm were not discussed in the study. Specific weaknesses in the selected classifiers, such as moderate accuracy levels and issues related to scalability and flexibility, may impact their suitability for real-world health care applications. Ultimately, some difficulties in applying this approach may emerge in ever-changing contexts if data scientists are not suitably trained in MCDM techniques.

In the study, criterion weights were determined by the IF-AHP method. The AHP method requires more evaluations than other weighting methods [98], such as the best-and-worst method, and it is difficult to detect inconsistent evaluations while evaluating. In addition, it was not tested whether a follow-up group representing all patients with PD was included for the study validation. It is recommended that researchers address these aspects in future studies.

## Abbreviations

AI: artificial intelligence
AT: assistive technology
CoCoSo: combined compromise solution
CR: consistency ratio
DEMATEL: decision-making trial and evaluation laboratory
FAHP: fuzzy analytic hierarchy process
IBK: instance-based k-nearest neighbors
IF-AHP: intuitionistic fuzzy analytic hierarchy process
IF-DEMATEL: intuitionistic fuzzy decision-making trial and evaluation laboratory
IFS: intuitionistic fuzzy set
IFWA: intuitionistic fuzzy weighted averaging
k-NN: k-nearest neighbors
MCDM: multicriteria decision-making
ML: machine learning
PD: Parkinson disease
SAW: simple additive weighting
TAM: Technology Acceptance Model
TOPSIS: technique for order of preference by similarity to ideal solution
UTAUT: Unified Theory of Acceptance and Use of Technology

## References

[1] GHE: life expectancy and healthy life expectancy. World Health Organisation.

[2] Hajat C, Stein E (2018). The global burden of multiple chronic conditions: a narrative review. Prev Med Rep.

[3] Cook EJ, Randhawa G, Sharp C (2016). Exploring the factors that influence the decision to adopt and engage with an integrated assistive telehealth and telecare service in Cambridgeshire, UK: a nested qualitative study of patient ‘users’ and ‘non-users’. BMC Health Serv Res.

[4] Ortiz‐Barrios M, Nugent C, Cleland I, Donnelly M, Verikas A (2020). Selecting the most suitable classification algorithm for supporting assistive technology adoption for people with dementia: a multicriteria framework. Multi Criteria Decision Anal.

[5] Ortíz-Barrios M, Cleland I, Donnelly M (2020). Digital Human Modeling and Applications in Health, Safety, Ergonomics and Risk Management Human Communication, Organization and Work.

[6] Ecer F (2022). An extended MAIRCA method using intuitionistic fuzzy sets for coronavirus vaccine selection in the age of COVID-19. Neural Comput Appl.

[7] Ecer F, Pamucar D (2021). MARCOS technique under intuitionistic fuzzy environment for determining the COVID-19 pandemic performance of insurance companies in terms of healthcare services. Appl Soft Comput.

[8] Ecer F, Böyükaslan A, Hashemkhani Zolfani S (2022). Evaluation of cryptocurrencies for investment decisions in the era of Industry 4.0: a Borda count-based intuitionistic fuzzy set extensions EDAS-MAIRCA-MARCOS multi-criteria methodology. Ax.

[9] Statistics. Parkinson’s Foundation.

[10] Venkatesh V, Davis FD (2000). A theoretical extension of the Technology Acceptance Model: four longitudinal field studies. Manag Sci.

[11] Shachak A, Kuziemsky C, Petersen C (2019). Beyond TAM and UTAUT: future directions for HIT implementation research. J Biomed Inform.

[12] Alwabel ASA, Zeng XJ (2021). Data-driven modeling of technology acceptance: a machine learning perspective. Exp Syst Appl.

[13] Chaurasia P, McClean SI, Nugent CD (2016). Modelling assistive technology adoption for people with dementia. J Biomed Inform.

[14] Chaurasia P, McClean S, Nugent CD (2022). Modelling mobile-based technology adoption among people with dementia. Pers Ubiquitous Comput.

[15] Ortíz-Barrios MA, Garcia-Constantino M, Nugent C, Alfaro-Sarmiento I (2022). A novel integration of IF-DEMATEL and TOPSIS for the classifier selection problem in assistive technology adoption for people with dementia. Int J Environ Res Public Health.

[16] Sharma R, Mishra R (2014). A review of evolution of theories and models of technology adoption. Indore Manag J.

[17] Zhang S, McClean SI, Nugent CD (2013). A predictive model for assistive technology adoption for people with dementia. IEEE J Biomed Health Inform.

[18] Stević Ž, Pamučar D, Puška A, Chatterjee P (2020). Sustainable supplier selection in healthcare industries using a new MCDM method: measurement of alternatives and ranking according to COmpromise solution (MARCOS). Comput Ind Eng.

[19] Mardani A, Jusoh A, MD Nor K, Khalifah Z, Zakwan N, Valipour A (2015). Multiple criteria decision-making techniques and their applications – a review of the literature from 2000 to 2014. Econ Res Ekon Istr.

[20] Parashar S, Bhattacharya S, Titiyal R, Guha Roy D (2024). Assessing environmental performance of service supply chain using fuzzy TOPSIS method. Health Serv Outcomes Res Method.

[21] Allah Bukhsh Z, Stipanovic I, Klanker G, O’ Connor A, Doree AG (2019). Network level bridges maintenance planning using Multi-Attribute Utility Theory. Struct Infrastruct Eng.

[22] Erdebilli B, Sicakyuz C, Yilmaz İ (2024). An integrated multiple-criteria decision-making and data envelopment analysis framework for efficiency assessment in sustainable healthcare systems. Healthcare Analytics.

[23] Rouyendegh BD, Oztekin A, Ekong J, Dag A (2019). Measuring the efficiency of hospitals: a fully-ranking DEA–FAHP approach. Ann Oper Res.

[24] Erol I, Oztel A, Searcy C, Medeni İT (2023). Selecting the most suitable blockchain platform: a case study on the healthcare industry using a novel rough MCDM framework. Technol Forecast Soc Change.

[25] Liu Y, Yang Y, Liu Y, Tzeng GH (2019). Improving sustainable mobile health care promotion: a novel hybrid MCDM method. Sustainability.

[26] Siksnelyte-Butkiene I, Zavadskas EK, Streimikiene D (2020). Multi-criteria decision-making (MCDM) for the assessment of renewable energy technologies in a household: a review. Energ.

[27] van Laarhoven PJM, Pedrycz W (1983). A fuzzy extension of Saaty’s priority theory. Fuzzy Sets Syst.

[28] Ben Rabia MA, Bellabdaoui A (2023). Collaborative intuitionistic fuzzy-AHP to evaluate simulation-based analytics for freight transport. Expert Syst Appl.

[29] Chen FH, Hsu TS, Tzeng GH (2011). A balanced scorecard approach to establish a performance evaluation and relationship model for hot spring hotels based on a hybrid MCDM model combining DEMATEL and ANP. Int J Hosp Manag.

[30] Lu MT, Lin SW, Tzeng GH (2013). Improving RFID adoption in Taiwan’s healthcare industry based on a DEMATEL technique with a hybrid MCDM model. Decis Support Syst.

[31] Bhattacharjee P, Howlader I, Rahman M (2023). Critical success factors for circular economy in the waste electrical and electronic equipment sector in an emerging economy: implications for stakeholders. J Clean Prod.

[32] Yilmaz I, Erdebilli B, Naji MA, Mousrij A (2023). A Fuzzy DEMATEL framework for maintenance performance improvement: a case of Moroccan chemical industry. J Eng Res.

[33] Abdullah L, Mohd Pouzi H, Awang NA (2023). Intuitionistic fuzzy DEMATEL for developing causal relationship of water security. IJICC.

[34] Büyüközkan G, Güleryüz S, Karpak B (2017). A new combined IF-DEMATEL and IF-ANP approach for CRM partner evaluation. Int J Prod Econ.

[35] Yazdani M, Zarate P, Kazimieras Zavadskas E, Turskis Z (2019). A combined compromise solution (CoCoSo) method for multi-criteria decision-making problems. MD (Chic).

[36] Panchagnula KK, Sharma JP, Kalita K, Chakraborty S (2023). CoCoSo method-based optimization of cryogenic drilling on multi-walled carbon nanotubes reinforced composites. Int J Interact Des Manuf.

[37] Torkayesh AE, Pamucar D, Ecer F, Chatterjee P (2021). An integrated BWM-LBWA-CoCoSo framework for evaluation of healthcare sectors in Eastern Europe. Socioecon Plann Sci.

[38] Tripathi DK, Nigam SK, Rani P (2023). New intuitionistic fuzzy parametric divergence measures and score function-based CoCoSo method for decision-making problems. Decis Mak Appl Manag Eng.

[39] Atanassov KT (1999). Intuitionistic Fuzzy Sets.

[40] Büyüközkan G, Göçer F (2017). Application of a new combined intuitionistic fuzzy MCDM approach based on axiomatic design methodology for the supplier selection problem. Appl Soft Comput.

[41] Atanassov KT (1996). An equality between intuitionistic fuzzy sets. Fuzzy Sets Syst.

[42] Ocampo LA (2019). Applying fuzzy AHP–TOPSIS technique in identifying the content strategy of sustainable manufacturing for food production. Environ Dev Sustain.

[43] Ocampo L, Yamagishi K (2020). Modeling the lockdown relaxation protocols of the Philippine government in response to the COVID-19 pandemic: an intuitionistic fuzzy DEMATEL analysis. Socioecon Plann Sci.

[44] Anzilli L, Facchinetti G (2016). Novel Developments in Uncertainty Representation and Processing.

[45] Karacan I, Senvar O, Arslan O, Ekmekçi Y, Bulkan S (2020). A novel approach integrating intuitionistic fuzzy analytical hierarchy process and goal programming for chickpea cultivar selection under stress conditions. Processes.

[46] Abdullah L, Najib L (2016). Sustainable energy planning decision using the intuitionistic fuzzy analytic hierarchy process: choosing energy technology in Malaysia. Int J Sustainable Energy.

[47] Ortíz-Barrios MA, Madrid-Sierra SL, Petrillo A, Quezada LE (2023). A novel approach integrating IF-AHP, IF-DEMATEL and CoCoSo methods for sustainability management in food digital manufacturing supply chain systems. JEIM.

[48] Boran FE, Genç S, Kurt M, Akay D (2009). A multi-criteria intuitionistic fuzzy group decision making for supplier selection with TOPSIS method. Expert Syst Appl.

[49] Saaty TL (2008). Decision making with the analytic hierarchy process. IJSSCI.

[50] Saaty TL, Greco S, Ehrgott M, Figueira J (2016). Multiple Criteria Decision Analysis International Series in Operations Research & Management Science.

[51] Mu E, Chung TR, Reed LI (2017). Paradigm shift in criminal police lineups: eyewitness identification as multicriteria decision making. Int J Prod Econ.

[52] Petrillo A, Colangelo F, Farina I, Travaglioni M, Salzano C, Cioffi R (2022). Multi-criteria analysis for life cycle assessment and life cycle costing of lightweight artificial aggregates from industrial waste by double-step cold bonding palletization. J Clean Prod.

[53] Fattoruso G, Barbati M, Ishizaka A (2024). An AHP parsimonious based approach to handle manufacturing errors in production processes. Prod Plan Control.

[54] Khan S, Haleem A (2021). Investigation of circular economy practices in the context of emerging economies: a CoCoSo approach. Int J Sustain Eng.

[55] Ecer F, Pamucar D (2020). Sustainable supplier selection: a novel integrated fuzzy best worst method (F-BWM) and fuzzy CoCoSo with Bonferroni (CoCoSo’B) multi-criteria model. J Clean Prod.

[56] Chen QY, Liu HC, Wang JH, Shi H (2022). New model for occupational health and safety risk assessment based on Fermatean fuzzy linguistic sets and CoCoSo approach. Appl Soft Comput.

[57] Ethics and approvals. UK Research and Innovation.

[58] Bot BM, Suver C, Neto EC (2016). The mPower study, Parkinson disease mobile data collected using ResearchKit. Sci Data.

[59] Greer J, Cleland I, McClean S (2018). Predicting assistive technology adoption for people with Parkinson’s disease using mobile data from a smartphone. https://www.worldscientific.com/worldscibooks/10.1142/11069.

[60] Hamad RA, Hidalgo AS, Bouguelia MR, Estevez ME, Quero JM (2020). Efficient activity recognition in smart homes using delayed fuzzy temporal windows on binary sensors. IEEE J Biomed Health Inform.

[61] Lee CH, Yoon HJ (2017). Medical big data: promise and challenges. Kidney Res Clin Pract.

[62] Endriyas M, Alano A, Mekonnen E (2019). Understanding performance data: health management information system data accuracy in Southern Nations Nationalities and People’s Region, Ethiopia. BMC Health Serv Res.

[63] Polat K, Nour M (2020). Parkinson disease classification using one against all based data sampling with the acoustic features from the speech signals. Med Hypotheses.

[64] Adler ED, Voors AA, Klein L (2020). Improving risk prediction in heart failure using machine learning. Eur J Heart Fail.

[65] Chang V, Bhavani VR, Xu AQ, Hossain MA (2022). An artificial intelligence model for heart disease detection using machine learning algorithms. Healthcare Analytics.

[66] Badillo S, Banfai B, Birzele F (2020). An introduction to machine learning. Clin Pharmacol Ther.

[67] Phillips B, Zhao H (1993). Predictors of assistive technology abandonment. Assist Technol.

[68] Miotto R, Wang F, Wang S, Jiang X, Dudley JT (2018). Deep learning for healthcare: review, opportunities and challenges. Brief Bioinf.

[69] Huang MW, Lin WC, Chen CW, Ke SW, Tsai CF, Eberle W (2016). Data preprocessing issues for incomplete medical datasets. Exp Syst.

[70] Chang C, Deng Y, Jiang X, Long Q (2020). Multiple imputation for analysis of incomplete data in distributed health data networks. Nat Commun.

[71] Lin WC, Tsai CF (2020). Missing value imputation: a review and analysis of the literature (2006–2017). Artif Intell Rev.

[72] Hasan MK, Alam MA, Roy S, Dutta A, Jawad MT, Das S (2021). Missing value imputation affects the performance of machine learning: a review and analysis of the literature (2010–2021). Inform Med Unlocked.

[73] Alabadla M, Sidi F, Ishak I (2022). Systematic review of using machine learning in imputing missing values. IEEE Access.

[74] Prince J, Andreotti F, De Vos M (2019). Multi-source ensemble learning for the remote prediction of Parkinson’s disease in the presence of source-wise missing data. IEEE Trans Biomed Eng.

[75] Sigcha L, Borzì L, Amato F (2023). Deep learning and wearable sensors for the diagnosis and monitoring of Parkinson’s disease: a systematic review. Exp Syst Appl.

[76] Harimoorthy K, Thangavelu M (2021). Cloud‐assisted Parkinson disease identification system for remote patient monitoring and diagnosis in the smart healthcare applications. Concurr Comput.

[77] Pecchia L, Bath P, Pendleton N, Bracale M, Tammy T AHP and risk management: a case study for assessing risk factors for falls in community-dwelling older patients.

[78] Pecchia L, Martin JL, Ragozzino A (2013). User needs elicitation via analytic hierarchy process (AHP). A case study on a computed tomography (CT) scanner. BMC Med Inform Decis Mak.

[79] Wang WC, Yu W der, Yang IT, Lin CC, Lee MT, Cheng YY (2013). Applying the AHP to support the best-value contractor selection-lessons learned from two case studies in Taiwan. J Civil Eng Manage.

[80] Schmidt K, Aumann I, Hollander I, Damm K, von der Schulenburg JMG (2015). Applying the analytic hierarchy process in healthcare research: a systematic literature review and evaluation of reporting. BMC Med Inform Decis Mak.

[81] Pereira RB, Plastino A, Zadrozny B, Merschmann LHC (2018). Correlation analysis of performance measures for multi-label classification. Inf Process Manag.

[82] Ali NA, El Abbassi A, Cherradi B (2022). The performances of iterative type-2 fuzzy C-mean on GPU for image segmentation. J Supercomput.

[83] Begio Y (2017). Yoshua Begio and Gary Marcus on the best way forward for AI. https://medium.com/@Montreal.AI/transcript-of-the-ai-debate-1e098eeb8465.

[84] Li J, Chang X (2021). Improving mobile health apps usage: a quantitative study on mPower data of Parkinson’s disease. ITP.

[85] Ozanne A, Johansson D, Hällgren Graneheim U, Malmgren K, Bergquist F, Alt Murphy M (2018). Wearables in epilepsy and Parkinson’s disease-a focus group study. Acta Neurol Scand.

[86] Cartuyvels R, Spinks G, Moens MF (2021). Discrete and continuous representations and processing in deep learning: looking forward. AI Open.

[87] Hoi SCH, Sahoo D, Lu J, Zhao P (2021). Online learning: a comprehensive survey. Neurocomputing.

[88] Raja PS, Thangavel K (2020). Missing value imputation using unsupervised machine learning techniques. Soft Comput.

[89] Lin WC, Tsai CF, Zhong JR (2022). Deep learning for missing value imputation of continuous data and the effect of data discretization. Knowl Based Syst.

[90] Farokhzadian J, Khajouei R, Hasman A, Ahmadian L (2020). Nurses’ experiences and viewpoints about the benefits of adopting information technology in health care: a qualitative study in Iran. BMC Med Inform Decis Mak.

[91] Belić M, Bobić V, Badža M, Šolaja N, Đurić-Jovičić M, Kostić VS (2019). Artificial intelligence for assisting diagnostics and assessment of Parkinson’s disease-a review. Clin Neurol Neurosurg.

[92] Khan B, Fatima H, Qureshi A (2023). Drawbacks of artificial intelligence and their potential solutions in the healthcare sector. Biomed Mater Devices.

[93] Javed AR, Fahad LG, Farhan AA (2021). Automated cognitive health assessment in smart homes using machine learning. Sustain Cities Soc.

[94] Lu J, Song E, Ghoneim A, Alrashoud M (2020). Machine learning for assisting cervical cancer diagnosis: an ensemble approach. Fut Gen Comput Syst.

[95] Chlap P, Min H, Vandenberg N, Dowling J, Holloway L, Haworth A (2021). A review of medical image data augmentation techniques for deep learning applications. J Med Imaging Radiat Oncol.

[96] Lin Z, Sinha S, Zhang W Towards efficient and scalable acceleration of online decision tree learning on FPGA.

[97] Ferreira PJS, Cardoso JMP, Mendes-Moreira J (2020). kNN prototyping schemes for embedded human activity recognition with online learning. Comp.

[98] Vega de la Cruz LO, Marrero Delgado F, Pérez Pravia MC (2022). Procedimiento para la gestión integrada del control interno con enfoque multicriterio [Article in Spanish]. IC.

